# A phase 2 study of trametinib for patients with pediatric glioma or plexiform neurofibroma with refractory tumor and activation of the MAPK/ERK pathway: TRAM-01

**DOI:** 10.1186/s12885-019-6442-2

**Published:** 2019-12-27

**Authors:** Sébastien Perreault, Valérie Larouche, Uri Tabori, Cynthia Hawkin, Sarah Lippé, Benjamin Ellezam, Jean-Claude Décarie, Yves Théoret, Marie-Élaine Métras, Serge Sultan, Édith Cantin, Marie-Ève Routhier, Maxime Caru, Geneviève Legault, Éric Bouffet, Lucie Lafay-Cousin, Juliette Hukin, Craig Erker, Nada Jabado

**Affiliations:** 1 Division of Child Neurology, Department of Pediatrics, CHU Sainte-Justine, Université de Montréal, 3175 Chemin de la Côte-Sainte-Catherine, Montreal, QC H3T 1C5 Canada; 2 Division of Hemato-Oncology, Department of Pediatrics, Centre Hospitalier Universitaire de Québec-Université Laval, Quebec City, QC Canada; 3 Division of Hemato-Oncology, Department of Pediatrics, Hospital for Sick Children, Toronto, ON Canada; 4 Department of Pathology, Hospital for Sick Children, Toronto, ON Canada; 5 CHU Sainte-Justine Research Center, CHU Sainte-Justine, Université de Montréal, Montreal, QC Canada; 6 Department of Pathology, CHU Sainte-Justine, Université de Montréal, Montreal, QC Canada; 7 Department of Radiology, CHU Sainte-Justine, Université de Montréal, Montreal, QC Canada; 8 Department of Pharmacology, CHU Sainte-Justine, Université de Montréal, Montreal, QC Canada; 9 Division of Neuropsychology, Centre Hospitalier Universitaire de Québec-Université Laval, Quebec City, QC Canada; 10 Division of Neurology, Department of Pediatrics, McGill University Health Center, Montreal Children’s Hospital, Montreal, QC Canada; 11 Departments of Oncology and Pediatrics, Alberta Children’s Hospital, University of Calgary, Cumming School of Medicine, Calgary, AB Canada; 12 Division of Child Neurology and Oncology, BC Children’s Hospital, University of British Columbia, BC, Vancouver, British Columbia Canada; 13 Division of Hemato-Oncology, Department of Pediatrics, IWK Health Centre, Dalhousie University, Halifax, NS Canada; 14 Division of Hemato-Oncology, Department of Pediatrics, McGill University Health Center, Montreal Children’s Hospital, Montreal, QC Canada

**Keywords:** Trametinib, Glioma, Plexiform neurofibroma, Neurofibromatosis type 1, BRAF, MEK inhibitor

## Abstract

**Background:**

Pediatric low-grade gliomas (PLGG) are the most frequent brain tumors in children. Up to 50% will be refractory to conventional chemotherapy. It is now known that the majority of PLGG have activation of the MAPK/ERK pathway. The same pathway is also activated in plexiform neurofibromas (PNs) which are low-grade tumors involving peripheral nerves in patients with neurofibromatosis type 1 (NF1). These lesions are known to be refractory to chemotherapy. Specific MEK inhibitors such as trametinib are now available and have been approved for other cancers harboring mutations in the MAPK/ERK pathway such as melanoma. We have observed significant responses to trametinib in patients with refractory PLGG in our institutions and results from the phase I study are promising. The treatment appears not only efficacious but is also usually well tolerated. We hypothesize that we will observe responses in the majority of refractory PLGG and PN treated with trametinib in this phase 2 study.

**Methods:**

The primary objective is to determine the objective response rate of trametinib as a single agent for treatment of progressing/refractory tumors with MAPK/ERK pathway activation. The TRAM-01 study is a phase II multicentric open-label basket trial including four groups. Group 1 includes NF1 patients with progressing/refractory glioma. Group 2 includes NF1 patients with plexiform neurofibroma. Group 3 includes patients with progressing/refractory glioma with KIAA1549-BRAF fusion. Group 4 includes other patients with progressing/refractory glioma with activation of the MAPK/ERK pathway. Eligible patients for a given study group will receive daily oral trametinib at full dose for a total of 18 cycles of 28 days. A total of 150 patients will be enrolled in seven Canadian centers. Secondary objectives include the assessment of progression-free survival, overall survival, safety and tolerability of trametinib, serum levels of trametinib and evaluation of quality of life during treatment.

**Discussion:**

Trametinib will allow us to target directly and specifically the MAPK/ERK pathway. We expect to observe a significant response in most patients. Following our study, trametinib could be integrated into standard treatment of PLGG and PN.

**Trial registration:**

ClinicalTrials.gov Identifier: NCT03363217 December 6, 2017.

## Background

### Pediatric low-grade gliomas

Pediatric low grade gliomas (PLGG) which include pilocytic astrocytoma (PA) are the most frequent brain tumors and represent 25–30% of central nervous system tumors in children [1]. While some patients can be cured with surgery alone, more than 70% need complimentary treatments due to the location of tumors that preclude resection [2]. Standard therapy for PLGG includes chemotherapy with a combination of intravenous carboplatin and vincristine, or weekly vinblastine for 70 weeks. Unfortunately, more than 50% of patients will have progressive disease despite conventional treatment(s) [3] [4]. Radiotherapy remains an option, but this approach has significant long-term side effects including cognitive dysfunction, endocrinopathies and vasculopathies [5]. Several clinical trials have focused on treatments of refractory PLGG but have failed to show significant efficacy and there is currently no standard therapy.

Recently, it has been found that the majority of PLGG have an activation of the MAPK/ERK pathway throughout various genetic mutations and alterations [6]. The signaling cascade culminates with ERK translocating to the nucleus, where it activates transcription factors that result in gene expression promoting growth and mitosis [7]. PLGG presents three major genetic alterations resulting in the activation of the MAPK pathway: NF1 mutation, BRAF fusion and BRAF mutation V600E [6].

NF1 mutations are mainly found in patients with neurofibromatosis type 1 (NF1). NF1 is one of the most frequent autosomal dominant diseases and affects 1 in 3000 individuals. Patients with NF1 have a susceptibility to develop tumor including plexiform neurofibroma (PN) and PLGG [8]. Up to 20% of NF1 patients will develop optic pathway glioma (OPG) and most of them will require treatment in order to preserve visual integrity [9]. NF1 patients can also develop PA in various locations such as the brainstem and subcortical areas [10].

The BRAF V600E mutation lies in the kinase domain and results in a constitutive activation of the MAPK/ERK pathway. The V600E mutation is positive in 5–10% of PA usually involving the brainstem and deep gray nuclei [11] [12].

The fusion between KIAA1549 (an uncharacterized gene) and the BRAF oncogene was reported to be a common feature of PA [13]. This fusion results in a constitutive activation of BRAF kinase activity with the loss of the BRAF N-terminal autoregulatory domain [14]. The KIAA1549:BRAF fusion is found in up to 77% of PA [15].

Finally, other mutations in PLGG were also found to activate the MAPK pathway through rare BRAF fusions or mutations, kinase domain duplications of FGFR1, and fusions of the NTRK gene (reviewed in Sturm et al., JCO 2017) [6, 16, 17]. Clinical implication of each mutation in terms of progression and response rate is currently unknown.

### NF1 with Plexiform Neurofibroma

Up to 50% of NF1 patients will develop plexiform neurofibromas (PNs) which affect large peripheral nerves [18, 19]. Despite distinctive histology and location of PNs when compared to PLGG, there is also an activation of the MAPK/ERK pathway through NF1 mutations.

PNs usually progress relentlessly during childhood, adolescence and adulthood causing lifelong disfigurement, disability, and mortality [18]. PNs can compress vital organs and create an array of morbidities.

Treatment of PNs consists primarily of symptoms management and/or surgical resection. However, the tumor’s close involvement with nerve, vasculature, or other viscera complicates surgery with ensuing frequently incomplete resection followed by tumor re-growth, or morbidity. Although several molecularly targeted compounds are in preclinical and clinical studies, but there is currently no approved medical therapy or cure for PNs.

### Targeting the MAPK/ERK pathway

Because of its key activation in PLGG, targeting the MAPK/ERK with small molecules offers new therapeutic possibilities.

Dabrafenib, a selective BRAF inhibitor, was shown to be effective in PLGG with BRAF V600E mutations [20]. Additionally, a phase II clinical trial with dabrafenib and trametinib for patients with PLGG and high-grade glioma with V600E mutation is underway (NCT02684058). However, patients that hold BRAF fusions treated with sorafenib alone had an acceleration of tumor growth likely related to paradoxical ERK upregulation [21]. As such, MEK inhibitors, which act further down in the molecular pathway, may be a better treatment alternative for these patients.

Recently, phase I and II study with selumetinib (another MEK inhibitor) showed promising antitumor activity in PLGG [22] [23] and Dombi et al. demonstrated dramatic responses in patients with PN treated with selumetinib [24]. However, this agent is still under investigation, is not available in Canada and has not been approved yet for treatment of PN or PLGG.

### Trametinib

Trametinib is a reversible, highly selective allosteric inhibitor of MEK1/MEK2 activation and kinase activity. It has good oral bioavailabily (72%). Food can decrease trametinib AUC by 24% and Cmax by 70%, so it is recommended to be administered under fasting conditions, either 1 h before or 2 h after a meal. It is highly bound to plasma proteins (97%). It is metabolized mainly via deacetylation alone (non-CYP mediated) or with mono-oxygenation in combination with glucuronidation. In vitro, trametinib is an inhibitor of CYP2C8, an inducer of CYP3A4 and a substrate of P-gp, but no significant drug interaction has been identified. It is mainly eliminated via the feces (≥ 80%), and to a lesser extent in urine (< 20%), mainly as inactive metabolites. Less than 0.1% of the parent drug is recovered in the feces and the urine. Ouellet et al. observed that trametinib oral clearance was lower in women compared to men (1.26-fold) and increased with body weight [25]. The half-life of trametinib is 5.3 days after a single dose administration and steady state is achieved by day 15. So far, all pharmacokinetics data are coming from adult studies, but there are ongoing studies evaluating pharmacokinetics data in pediatric populations. Available formulations and strengths are trametinib 0.5 mg tablets, trametinib 2 mg tablets and trametinib powder for oral solution (0.05 mg/mL). Cox et al. evaluated the bioavailability of the oral solution compared with the tablet formulation. They found similar AUCs, but the Cmax of the oral solution was higher and the Tmax earlier compared to the tablet formulation [26]. In Canada, trametinib was approved as monotherapy in 2013 and in combination with dabrafenib in 2016 for the treatment of adult patients with unresectable or metastatic melanoma with a BRAF V600E mutation [27] [28].

Grossauer et al. demonstrated the efficacy of trametinib in murine xenografts with V600E high grade glioma [12]. Recently, Geoerger et al. presented results of their phase I for safety and tolerability of trametinib in pediatric patients with refractory solid tumors [29]. Overall, trametinib was well tolerated with relatively few side effects. Among patients treated with trametinib monotherapy, hyponatremia (*n* = 2) and pyrexia (n = 2) were the only treatment-related serious adverse events (SAEs) reported in > 1 patients. The recommended dose was 0.025 mg/kg daily for patients of 6 years and older and 0.032 mg/kg for patients younger than 6 years old. This study was not designed to assess efficacy of trametinib but it was reported that in 40 of the patients with LGG or NF1-related PN, 7 showed partial response (PR), 21 patients had stable disease (SD) and only 4 had progressive disease (PD). Seven patients were not evaluable. It is not reported at this date how many patients with SD were considered having a minor response (MR) (decrease in lesion size of > 25% to ≤50%).

In case reports, tumors with KIAA1549:BRAF fusion were found to be very sensitive to trametinib [30]. In our series of six patients treated with trametinib, one showed PR, four had MR and only one had PD [31]. Our observations are in line with what Geoerger reported in their phase I study [29].

### Study rationale

Since there is no standard treatment for refractory PLGG and limited conventional chemotherapy regimens, we developed a study to target the MAPK/ERK pathway. Trametinib was selected because of its bioavailability, long half-life and availability in a liquid oral solution (currently only available for clinical trial). This drug has also been used on a compassionate basis for PLGG patients over the years in our centers. While designing this study, we included a specific group for NF1 patients with PN since treatment with trametinib is also promising for this population. A classical randomized clinical trial could not be conducted since there is no standard efficacious second-line treatment and the use of a placebo would not be ethical in this situation. We therefore designed an open label modified phase II basket trial with four patient groups.

## Methods/design

### Objectives

The primary objective is to determine the response rate of daily trametinib as a single agent for treatment of progressing/refractory tumors with MAPK/ERK pathway activation. The response rate is defined as the proportion of patients with stable disease (SD), minor response (MR), partial response (PR) and complete response (CR) as the best response on study.

Secondary objectives include:

- Determine efficacy outcome defined as time to progression (TTP), progression free survival (PFS) and overall survival (OS) up to three years following completion of treatment.
- Determine the safety and tolerability of trametinib. Adverse events (AE) and severe adverse events (SAE) will be monitored.
- Analyse the serum levels of trametinib. Analysis of trough level will be done twice (at day 22, and before starting cycle 16 or at progression whichever comes first).
- Assess changes in quality of life over treatment phases and compare pre and post changes across groups. Evaluations of quality of daily life will be recorded at inclusion and every six months with the Pediatric Quality of Life Inventory (PedsQL) (Generic Core Scales/Brain tumor modules/Infant Scales).

Exploratory objectives include:

- Determine if there are cognitive changes in patients with NF1 during treatment with trametinib. Neurocognitive assessment will be performed at inclusion and at completion of treatment using: Bayley-III, D-KEFS, WPPSIV, WISC-V and WAIS-IV depending on the age of patients with NF1.
- To investigate and correlate biological features to tumor response. Gene expression profiling will be done on fresh frozen tissue and mutational analysis on paraffin-embedded tissue. Circulating tumor DNA in blood (ctDNA) will be assessed throughout treatment.

### Study design

The TRAM-01 study is a phase II open-label basket trial. A total of seven pediatric academic hospitals will be participating. Four groups of patients will be included. Group 1 includes NF1 patients with progressing/refractory glioma. Group 2 includes NF1 patients with plexiform neurofibroma. Group 3 includes patients with progressing/refractory glioma with KIAA1549-BRAF fusion. Group 4 includes other patients with progressing/refractory glioma with activation of the MAPK/ERK pathway. Mutational status is determined locally in participating institutions and will be confirmed centrally.

### Ethical consideration

Full ethical approval for the study was obtained from the Research Ethics Committee from CHU Sainte-Justine. The TRAM-01 study will be conducted according to the principles of the Declaration of Helsinki. Written consent to participate will be obtain from participant or parents/legal guardians for minors. Data management, monitoring and reporting of the study will be performed in accordance with the ICP-GCP guidelines.

### Inclusion criteria

1. Consent. Signed written informed consent prior to study participation
2. Assent. Assent from minor participants will be sought
3. Study activities compliance. Participants must be willing and able to comply with scheduled visits, treatment schedule, laboratory testing, and other requirements of the study, including disease assessment by contrast-enhanced MRI
4. Age. Patients must be aged ≥1 month (corrected age) to ≤25 years at the time of study enrollment
5. Study group. Participants must belong to one of the following groups to be eligible
  Group 1: NF1 with progressing/refractory LGG
  Group 2: NF1 with PN
  Group 3: Progressing/refractory LGG with KIAA1549-BRAF fusion.
  Group 4: Progressing/refractory glioma with activation of the MAPK/ERK pathway who do not meet criteria for other study groups.
6. Tumor Tissue. Sample Tumor tissue will be required for all patients (fresh tissue recommended when available). Patients with NF1 and LGG or PN can still be enrolled without tissue if no surgery or biopsy was conducted.
7. Previous MRI. At least two previous MRI for Group 1, 3, 4 and one previous MRI for Group 2 must be available for central review.
8. Prior therapy. Participants must have failed at least one line of treatment including chemotherapy and/or radiation therapy except for plexiform neurofibroma (since there is no recognized standard treatment for this tumor).
9. Prior therapy toxicity. Patients must have recovered to grade ≤ 1 from acute toxic effects of all prior chemotherapy, immunotherapy or radiotherapy prior to enrollment. Toxicities will be graded as per the National Cancer Institute Common Terminology Criteria for Adverse Events (CTCAE), version 5.0.
10. Prior therapy timeline. Participants having previously received a chemotherapy agent(s) and/or radiation must conform to the timeline described below. There is no limitation on the number of previous treatments or cycles received.
  An interval of at least 28 days after the last dose of a myelosuppressive chemotherapy, and at least 42 days after the last dose of Nitrosoureas is required prior to starting trametinib.
  An interval of at least 28 days after the last dose of any biologic agents including monoclonal antibody treatment, immunotherapy, viral therapy and other investigational agent is required prior to starting trametinib.
  An interval of at least 84 days after the end of radiation therapy is required prior to starting trametinib.
  An interval of at least 48 h for short-acting colony stimulating factor agents and 10 days interval for long-acting colony stimulating factor agents are required prior to starting trametinib.
11. Life expectancy. Patients must have a life expectancy of greater than 6 months.
12. Performance level. Patients must have a performance status corresponding to a Lansky/Karnofsky score ≥ 50.
13. Organ Function Requirements.

Participants must have normal organ and marrow function as defined below:

- Total leukocytes ≥3000/μL
- Absolute neutrophil count (ANC) ≥ 1000/μL
- Hemoglobin > 80 g/l (transfusion independent within last 2 weeks)
- Platelet count ≥100,000/μL (transfusion independent within last 2 weeks)
- Total bilirubin ≤1.5 times the upper limit of normal (ULN) within normal institutional limits for age
- Alanine Aminotransferase (ALT) ≤ 2.5 times the upper limit of normal (ULN)
- Serum creatinine within normal institutional limits for age OR creatinine clearance ≥60 mL/min/1.73 m^2^ for participants with creatinine levels above institutional normal.
- Creatine phosphokinase ≤2x ULN
- A cardiac function defined as Corrected QT (QTcB) interval < 480 msec and LVEF ≥ lower limit of normal (LLN) by echocardiogram (ECHO).
- Blood pressure must be smaller or equal to the 95th percentile for patient’s age, height and gender.

14. Reproductive status. Children of childbearing and child-fathering potential must agree to use adequate contraception. Males and females treated or enrolled in this protocol must also agree to use adequate contraception prior to the study, for the duration of study participation, and 6 months after completion of trametinib administration.
15. Administration of oral medication. Patients must be able to ingest and retain enterally (per os, nasogastric tube or gastrostomy) administered medication and be free of any clinically significant gastrointestinal abnormalities limiting the absorption of the medication. Tablets cannot be crushed. If the patient cannot swallow tablets, the liquid form should then be used.

### Exclusion criteria

1. Other investigational agents. Patients who are receiving any other investigational agents.
2. Cardiac exclusion criteria. Patient who has an ejection fraction inferior to the institution LLN, a QTcB ≥480 msec or an absolute resting left ventricular ejection fraction (LVEF) of ≤39% are not eligible for enrolment.
3. Presence of another malignancy. Patient has any other malignancy except if the other primary malignancy is neither currently clinically significant nor requiring active intervention
4. Previous MEK inhibitor treatment. Participants previously treated with a MEK inhibitor who showed less than stable disease during treatment
5. Tumor with BRAF V600E mutation. Patients with a tumor presenting a positive BRAF V600E mutation
6. Other uncontrollable medical disease. Patient who has a severe and uncontrollable medical disease, has a chronic liver disease, uncontrolled intercurrent illness including, but not limited to, ongoing or active infection, symptomatic congestive heart failure, unstable angina pectoris, cardiac arrhythmia, or psychiatric illness/social situations that would limit compliance with study requirements
7. Known HIV infection. Patient who has a known diagnosis of human immunodeficiency virus (HIV) infection, hepatitis B or C
8. Previous surgery. Patients who had major surgery within 2 weeks prior to study entry
9. Allergy. History of allergic reactions attributed to compounds of similar chemical or biologic composition to trametinib
10. Previous history of non-compliance. Patients with a previous significant history of non-compliance to their treatment or medical regimen
11. Pregnant or breastfeeding patients. Pregnant or breastfeeding female patients are not eligible for this study

### Sample size

We expect to recruit a total of 150 (Group 1 and 3 = 42 patients each, Group 2 = 46 patients and Group 4 = 20 patients). Sample size was calculated based on the assumption that trametinib will be considered inactive if the true response rate is 40% or less; however, if the true response rate is 60% or more, then this treatment would be considered worthy of further study. Therefore, set H0: response rate = 0.40 (lower limit) versus HA: response rate = 0.60. Simon optimal model was used. For Groups 1, 2 and 3 a minimum of 39 patients is needed in order to support or reject H0. Since 42 patients will be enrolled in Groups 1 and 3 and 46 patients in Group 2 this will account for non-compliance and loss to follow-up of 7 and 15% respectively. Group 4 will be looking at the feasibility to include and treat patients without NF1 and KIAA1549-BRAF fusion. If the recruitment cannot be completed for any reason, participants in Groups 1, 3 and 4 can be pooled to test H0 for PLGG.

### Intervention

During the treatment phase, patients will receive trametinib at a fixed dose of 0.025 mg/kg (patients ≥6 years) or 0.032 mg/kg (patients < 6 years) with no dose escalation. Trametinib will be administered for up to 18 cycles of 28 days to assess the efficacy and safety in patients with PLGG or PN. Patients weighting < 33 kg or who cannot swallow tablets will receive the oral solution. Available formulations and strengths are trametinib 0.5 mg tablets, trametinib 2 mg tablets and trametinib powder for oral solution (0.05 mg/mL). For toxicity related to trametinib, one dose reduction will be accepted. The need for a second dose reduction will lead to the permanent discontinuation of study treatment. Specific guidelines are available in the protocol for management of toxicity.

### Follow-up phase

Patients will be followed every six months for up to 3 years post-treatment or progression. They will be followed for overall survival, further progression and information on further lines of anti-cancer treatments; if known, dates of initiation and end dates will be collected.

### Data collection

All data for the TRAM-01 study is entered in a customized Electronic Data Capture system developed by Information Management Systems (IMS) at the Lady Davis Institution.

### Radiological evaluation

MRI will be done at screening and every 3 cycles during treatment phase and every six months during the follow-up period.

Baseline lesions and responses for Groups 1, 3 and 4 will be evaluated using the modified Response Assessment in Neuro-Oncology (RANO). Target lesion(s) must measure at least 10 mm by 5 mm.

- Complete Response (CR) - No radiological evidence of tumor on MRI scans.
- Partial Response (PR) - Greater than 50% reduction in the sum of the product of the greatest tumor diameter and its perpendicular diameter taking as reference the baseline measurements by MRI scan.
- Minor Response (MR) - 25-50% reduction in the sum of the product of the greatest tumor diameter and its perpendicular diameter taking as reference the baseline measurements by MRI scan.
- Stable Disease (SD) - Less than a 25% decrease or ≤ 25% increase in the sum of the product of the greatest tumor perpendicular diameters taking as reference the smallest measurement since treatment started by MRI scan.
- Progressive Disease (PD) - Greater than a 25% increase in the sum of the product of the greatest tumor perpendicular diameters in the tumour size by MRI taking as reference the smallest measurement since treatment started by MRI scan, or appearance of one or more new tumoral lesion in a previously uninvolved area on MRI scan.

Baseline lesions and responses for Group 2 will be evaluated using a modified Response Evaluation Criteria in Solid Tumors (RECIST 1.1). Target lesion(s) must measure at least 30 mm in on direction.

- Complete Response (CR)- No radiological evidence of tumor on MRI scans.
- Partial Response (PR) - Greater than 50% reduction in the sum of the greatest tumors diameters on MRI scan taking as reference baseline measurements.
- Minor Response (MR) - 25-50% reduction in the sum of the greatest tumors diameters on MRI scan taking as reference baseline measurements.
- Stable Disease (SD) - Less than a 25% decrease or ≤ 25% increase in the sum of the greatest tumors diameters taking as reference the smallest measurement since treatment started.
- Progressive Disease (PD) - Greater than a 25% increase in the sum of the greatest tumors diameters taking as reference the smallest measurement since treatment started or appearance of one or more new tumoral lesion in a previously uninvolved area.

All treatment responses throughout this study will be centrally reviewed by an independent radiologist.

### Neuropsychological evaluation

Neuropsychological evaluations will be conducted to assess cognitive function at start of treatment +/− 28 days, and at the end of treatment (between cycle 17 and the end of cycle 18) for NF1 patients (Groups 1 and 2 patients only). Testing battery will depend on age at inclusion (Bayley III < 3 years old, WPPSIV ≥3 to 5 years old, WISC-V ≥ 6 to 16 years old, WAIS-IV for ≥17 years old and D-KEFS for ≥8 years old).

### Quality of life evaluation

To document the quality of life of patients, the PedsQL Generic scale and Brain Tumor module [32–34]. The measures are available over the age span with an infant scale for patients under 2 years will be used to assess the physical, mental, social health dimensions, as well as the cognitive development of children [35]. The PedsQL integrates the generic and disease-specific approaches with child self-reports and parent proxy-reports [36]. Thus, both questionnaires will be completed by the patient and one caregiver.

### Biological study

To determine study group, the NF1 diagnosis will be confirmed clinically based on NIH criteria or by genetic testing. KIAA1549-fusion will be confirmed by FISH or CGH in local institution. V600E mutation will be excluded by immunohistochemistry staining or RT-PCR. Next generation sequencing will be done in the form of RNAseq and as needed a targeted panel (if no alteration is identified in RNAseq). All genetic alterations will be validated. DNA methylation assay using the 850 K array will be performed.

### Statistical analysis

A two-stage assessment during recruitment will be conducted.

For Groups 1 and 3, assuming alpha = 0.05 and power = 0.70:

During Stage 1, 11 patients will be accrued. Group will continue to Stage 2 if ≥6 patients have an objective response (total of best responses = SD + MR + PR + CR). During Stage 2, if ≤21 patients have an objective response, the treatment will be deemed inactive.

For Group 2, assuming alpha = 0.05 and power = 0.80:

During Stage 1, 16 patients will be accrued. Group will continue to Stage 2 if ≥8 patients have an objective response (total of best responses = SD + MR + PR + CR). During Stage 2, if ≤23 patients have an objective response, the treatment will be deemed inactive.

Objective responses will be listed by patient and timepoint; best response on study, TTP, PFS, and OS will be reported by patient. Descriptive summary statistics for each group will be presented for TTP, PFS, and OS. Curves for PFS, TTP, and OS may be estimated using Kaplan-Meier methods.

### Safety

Scientific evaluation was done at Sainte-Justine by an independent committee prior to submission to research ethics board (REB). The first ethical review was conducted at CHU Sainte-Justine.

An independent and outside ARO (academic research organization), Exactis Innovation, has been assigned for the management of this study. Exactis will be responsible for overseeing the regulatory aspects and, monitoring of sites, verify compliance and conduct site visits.

We will record all AEs and SAEs to better evaluate tolerability. The descriptions and grading scales found in the revised CTCAE version 5.0 will be used. Management of AEs of special interest is well described in the protocol. In order to detect early signs of toxicities, surveillance exams will include regular ophthalmologic evaluations, EKG, echocardiogram and X-ray of the growth plate. All SAEs must be reported within 24 h after the Investigator is made aware.

A Data Safety Monitoring Board (DSMB) was created following the study approval. DSMB will be tasked with determining safe and effective conduct of the study and with recommending the date for the conclusion of the trial based upon whether significant benefits or risks have developed. All members composing the DSMB are independent from the research team and free of any conflicts of interest.

A Data Monitoring Committee (DMC) will receive and review the progress and accrual data of this trial and will safeguard the interests of trial participants, periodically review and evaluate the accumulated study data for participant safety and efficacy and monitor the progress and overall conduct of the clinical trial. The DMC has access to quarterly study reports, raw study data so that they can see any emerging risks such as frequent or severe adverse events.

## Discussion

Based on ongoing phases I and II trial with MEK inhibitors and case series, trametinib is a potential efficacious therapy for PLGG with activation of the MAPK/ERK [30, 31] [29]. We designed this study to confirm trametinib efficacy, safety and assess the duration of response once the trametinib is stopped. There is currently no other similar or competing clinical trial for patients with PLGG. In fact, this is the first and only phase II clinical trial to use trametinib as a single agent for low grade glioma and/or plexiform neurofibroma. We decided to subdivide our glioma cohort into three distinct groups since response rate might vary depending on the molecular alteration. For example, NF1 patients might present a better response to trametinib compared to KIAA1549-BRAF fusion patients. Our group 4 includes patients with a MAPK/ERK activation (not NF1, KIAA1549-BRAF, or BRAF V600E) who could benefit from a MEK inhibitor. This group can include patients with KRAS mutation, rare fusion or BRAF mutation for example. This group will be heterogeneous in terms of molecular profile and will be exploratory.

Patients with NF1 and a plexiform neurofibroma are also likely to respond to trametinib. These patients will be included in a specific subgroup. However, dosing, duration of treatment, surveillance and management of side effects are essentially the same than in patients with glioma and this population was therefore included in this study.

During this study, we will evaluate not only standard response and toxicity, but we also include important outcomes such as quality of life. Indeed, late psychological-related effects have been demonstrated in pediatric low-grade gliomas, such as the reduction of health-related quality of life (HRQOL) [37]. Surveillance of HRQOL during treatments is essential, especially as brain tumor patients have reported a poor HRQOL [34]. In our experience, daily oral trametinib is better tolerated than weekly vinblastine or vincristine/carboplatin regimen.

Neurocognitive assessment of patients with NF1 receiving trametinib will be conducted. Based on our experiences we believe that young patients with NF1 might show improvement in their development milestones while receiving trametinib. Our study is not powered to specifically answer this hypothesis but these investigations may give us data supporting a future clinical trial dedicated to this important issue.

Finally, molecular analysis will allow us to better understand why most patients respond to treatment whereas a minority progress. Gliomas with specific mutations or methylation profiles might have better and more sustained response to trametinib.

## Data Availability

The data that support the findings of this study are available from the authors but restrictions apply to the availability of these data, which were used under license for the current study, and so are not publicly available. Data are however available from the authors upon reasonable request and with permission of Novartis.

## Appendix I

**Planned Participating centres with local investigator in alphabetical order.**

Alberta Children’s Hospital, Calgary, AB (Lucie Lafay-Cousin MD).

BC Children’s Hospital, Vancouver, BC (Juliette Hukin MD).

CHU Sainte-Justine, Montréal, QC (Sébastien Perreault MD).

CHU de Québec, Québec City, QC (Valérie Larouche MD).

Hospital for Sick Children’s-SickKids, Toronto, ON (Eric Bouffet MD).

IWK Health Centre, Halifax, NS (Craig Erker MD).

McGill University Health Centre, Montreal, QC (Geneviève Legault MD).

### Appendix II

Support and funding

Trametinib provided by Novartis

Funding

CIHR (Canadian Institutes of Health Research)

Fondation des Étoiles

Leucan

Fondation Charles Bruneau

Fondation Maurice Tanguay

Westmount Old Timers

Défi ski Banque National 2018

BMO Casino 2018

## Abbreviations

AE: Adverse events
CR: Complete response
ctDNA: Circulating tumor DNA in blood
DMC: Data Monitoring Committee
DSMB: Data Safety Monitoring Board
HRQOL: Health-related quality of life
LLN: Lower limit of normal
MR: Minor response
NF1: Neurofibromatosis type 1
OS: Overall survival
PA: Pilocytic astrocytoma
PedsQL: Pediatric Quality of Life Inventory
PFS: Progression free survival
PLGG: Pediatric low-grade gliomas
PR: Partial Response
QTcB: Corrected QT
SAE: Severe adverse events
SD: Stable disease
TTP: Time to progression
